# A Personal Electronic Health Record: Study Protocol of a Feasibility Study on Implementation in a Real-World Health Care Setting

**DOI:** 10.2196/resprot.6314

**Published:** 2017-03-02

**Authors:** Dominik Ose, Aline Kunz, Sabrina Pohlmann, Helene Hofmann, Markus Qreini, Johannes Krisam, Lorenz Uhlmann, Christian Jacke, Eva C Winkler, Hans-Joachim Salize, Joachim Szecsenyi

**Affiliations:** ^1^ Department of General Practice and Health Services Research University Hospital Heidelberg Heidelberg Germany; ^2^ Health System Innovation and Research Department of Population Health Sciences University of Utah Salt Lake City, UT United States; ^3^ Ethics and Patient-Oriented Care National Centre for Tumor Diseases (NCT) Heidelberg Germany; ^4^ Institute for Medical Biometry and Informatics Department of Medical Biometry University Hospital Heidelberg Heidelberg Germany; ^5^ Central Institute of Mental Health Medical Faculty Mannheim Heidelberg University Heidelberg Germany

**Keywords:** self-efficacy, personal electronic health record, colorectal cancer, chronic care, interprofessional collaboration, telemedicine, feasibility studies

## Abstract

**Background:**

A significant potential for patient empowerment is seen in concepts aiming to give patients access to their personal health information (PHI) and to share this PHI across different care settings and health systems. Personal health records (PHRs) and the availability of information through health information exchanges are considered to be key components of effective and efficient health care. With tethered PHRs, as often used in the United States, patients’ opportunities to manage their PHI are strongly restricted. Therefore, within the INFOPAT (information technology for patient oriented care) project (2012-2016) in Germany, funded by the Federal Ministry of Education and Research (BMBF), the development of a patient-controlled “personal electronic health record” (PEPA) was based on user requirements right from the beginning.

**Objective:**

The overall objective of the study is to implement and evaluate a PEPA prototype for patients with colorectal cancer who are treated at the National Center for Tumor Diseases in Heidelberg. To achieve this aim, this study has 2 parts: a pre-implementation study (phase 1) and an implementation study (phase 2). The pre-implementation study will include a usability evaluation of the PEPA approach and the consideration of organizational preconditions for the implementation. With the implementation study, we will evaluate the process of implementation (eg, barriers or facilitators), the need for organizational change (eg, processes of communication), and the impact on outcomes (eg, self-efficacy, involvement in care).

**Methods:**

The pre-implementation study is based on a mixed methods approach and comprises qualitative and quantitative element according to our research aim. We will use a think-aloud method for the usability analysis. Additionally, participants will be asked to evaluate their overall satisfaction based on a standardized questionnaire, the System Usability Scale. For the analysis of preconditions, we will conduct semistructured personal interviews with, for example, patients, medical assistants, and physicians. Within the implementation study the outcome evaluation is planned as a prospective, 3-month, open-label “before and after” trial. Additionally, for the analysis of processes and the need for organizational change, we will conduct interviews with the participants (eg, patients, general practitioners, physicians) of the before and after trial.

**Results:**

This project is part of the INFOPAT project, which is funded (2012-2016) by the Federal Ministry of Education and Research (BMBF). The enrolment was completed in July 2016. Data analysis is currently under way and the first results are expected to be submitted for publication at end of 2017.

**Conclusions:**

Existing approaches of PHRs aim to give patients access to their treatment data. With the PEPA approach and this study, we go a step further: patients have access to their PHI and they can give other persons (eg, their general practitioner) access. With this approach, new possibilities for professional collaboration and the engagement of patients can arise.

## Introduction

If patients are being treated across various health care settings or health systems, having access to their complete personal health information (PHI) can be problematic. This can lead to inefficiencies and may hinder coordination and continuity of care [1,2]. However, the health information exchange between different care settings and health systems is a key issue for a multidisciplinary approach in chronic care [3]. Additionally, a significant potential for patient empowerment is seen in concepts aiming to give patients access to their own health- and treatment-related data [4-6]. In particular, personal health records (PHRs) and the availability of information through health information exchange are considered to be key components of effective and efficient health care [7-9].

PHR systems, as often used in the United States, allow patients to access primary data from a provider-managed electronic health record through a patient portal (tethered PHRs). With these PHRs, patients’ opportunities to manage and to share their health information in cross-sectoral care are nevertheless restricted. In order to promote a more active patient role, it is important to empower patients to take more responsibility and participate actively in their health care. This may include controversial aspects such as allowing patients to decide which physician or other health care professional (HCP) gets access to their PHI during the course of treatment [10,11].

However, design and implementation of PHRs have not proven to be easy. Experiences from a nationwide implementation of PHRs (HealthSpace, England) have shown that they can fail because of a lack of alignment with users’ expectations and self-management practices [12]. According to adoption and use, the participation of patients and users (eg, physicians, other HCPs) in the development, implementation, and evaluation of innovative PHR approaches is central [8,12].

Within the INFOPAT project (2012-2016) in Germany, funded by the Federal Ministry of Education and Research (BMBF), the development of a patient-controlled “personal electronic health record” (PEPA) was based on user requirements right from the beginning (first study phase) [13]. The technical PEPA development is based on established health information technology standards and, in particular, “Integrating the Healthcare Enterprise.” As a subset of PHR, the Web-based PEPA would enable patients to access, maintain, and manage (including access management) a secure copy of their PHI from various primary systems of service providers (eg, electronic medical record in hospital, electronic health record in general practice).

**Figure 1:**
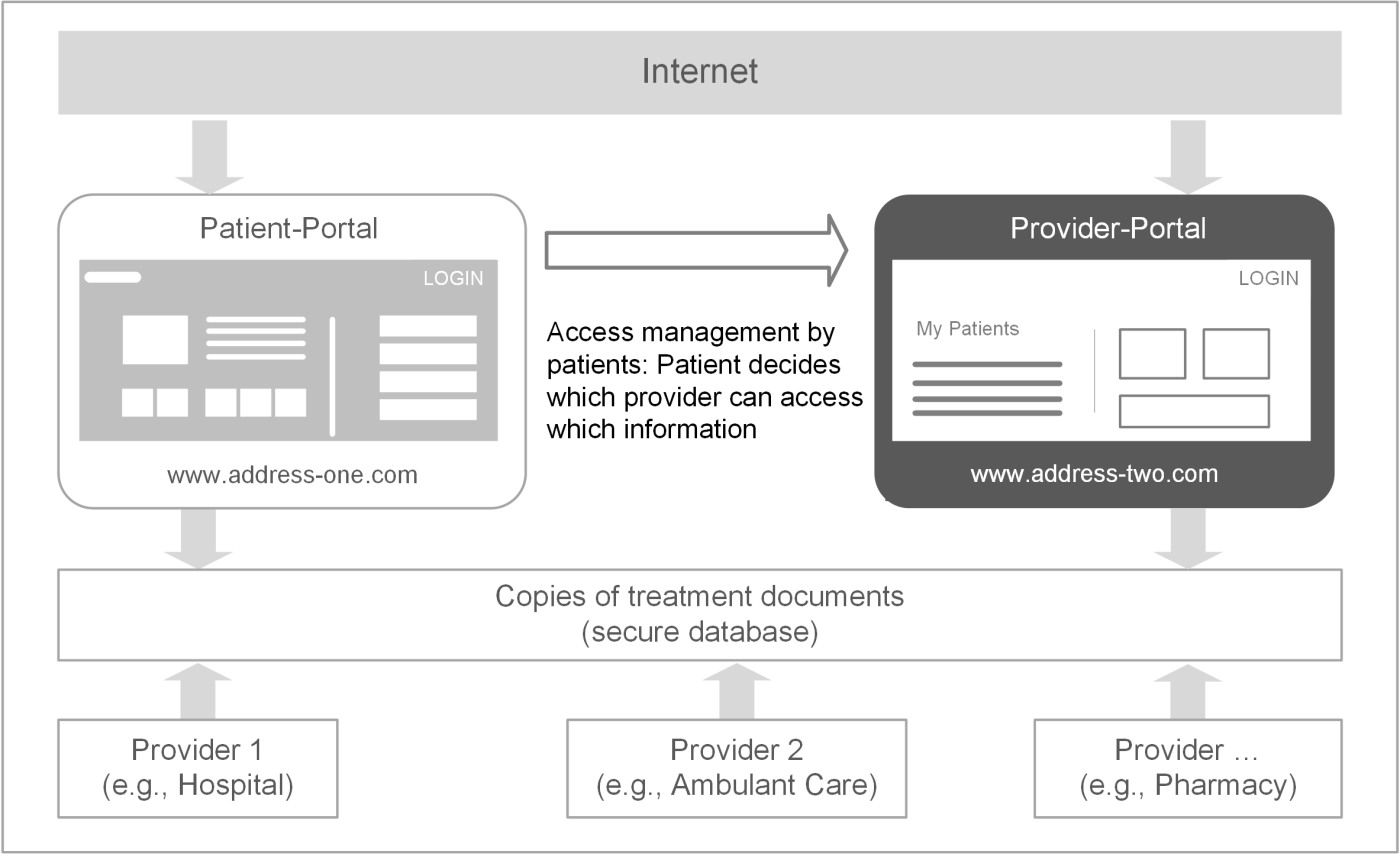
The PEPA (personal electronic health record) approach.

The PEPA concept comprises 2 Web-based portals, one for patients (“patient portal”) and one for HCPs (“professional portal”). Patients can log in to the patient portal and gain insight about their PHI. For managing the access to PEPA, the patient can decide in detail which HCP is able to access which PHI via the professional portal (Figure 1, adapted from [10]).

## Methods

Since the development of the PEPA proceeded [8-10], as a next step of the INFOPAT project the current version of the prototype will be brought into use for the first time. In order to do this, our feasibility study (ISRCTN: 85224823) for the implementation in a real-world health care setting is divided into 2 parts: pre-implementation (phase 1) and implementation (phase 2). Both phases will be described in detail in the following sections.

### Phase 1: Pre-implementation Study

When bringing new technologies into daily practice, it is inevitable that challenges will arise from system immanent conditions. If an implementation strategy does not focus on problems that patients and HCPs experience in their everyday life, it is doomed to fail. For that reason, it is envisaged to perform a pre-implementation study focusing on those factors that are crucial for implementation success or failure before determining the underlying implementation procedure.

**Figure 2:**
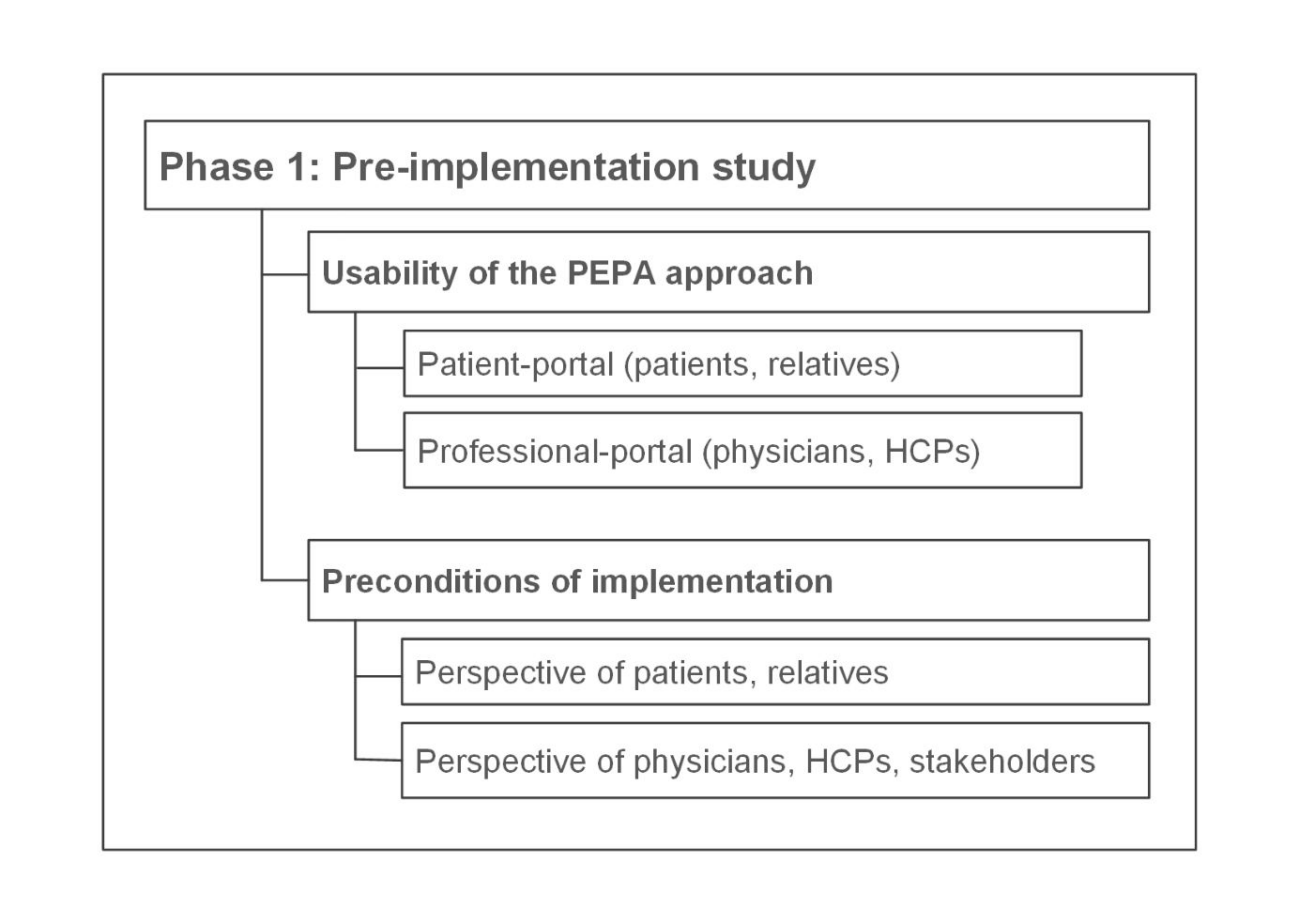
Parts of the pre-implementation study. PEPA: personal electronic health record; HCP: health care professional.

With the findings of the pre-implementation study it will be possible to improve the prototype and to create a catalog of requirements that addresses patients’ and professionals’ needs for PEPA usage as well as the surrounding conditions of the care setting. The catalog will serve as a precondition for the planned implementation of the PEPA. The pre-implementation study comprises 2 parts: the usability evaluation of the PEPA approach and the analysis of preconditions for implementation (Figure 2).

#### Objectives

##### Usability of the PEPA Approach

As described below (see Figure 1), the PEPA approach comprises 2 separate portals: patient portal (prototype) and professional portal (existing product). The usability evaluation of the patient portal aims to identify factors that may have an influence on the transfer of the prototype to the real-world health care setting. Those factors could be, for example, either issues of usability or other patient-perceived implementation barriers. Therefore, the usability evaluation of the patient portal places emphasis especially on unique features that have been identified as crucial for patients’ benefit of using a PHR [11,13]. The focus is on functionalities such as patient-controlled access to the PEPA, patient-controlled information exchange between HCPs, and patient-controlled data storage within the PEPA.

In contrast to the patient portal, the professional portal is not a prototype but an existing product that has already been used before in another health care environment. The usability evaluation will help to understand whether the professional portal can be integrated adequately into the PEPA context. In this context, relevant features are (1) the easy log-in and log-out, (2) the automatic data transmission, (3) interprofessional cooperation and communication, and (4) the manual upload of documents.

Overall, the research question for the usability evaluation of the PEPA approach is as follows: Do the patient portal and the professional portal meet the demands of their users regarding design, functionality, and usage? Answering this question includes the following objectives: to (1) point out specific challenges that arise during the testing of the PEPA approach and (2) to map demands for a training program and further development.

##### Preconditions of Implementation

The evaluation of preconditions aims to analyze potential barriers within the organizational or personal conditions of all user groups (patients, relatives, physicians, HCPs, stakeholders) and necessary requirements concerning transfer of the PEPA approach to a real-world health care setting. The analysis of preconditions will be helpful to derive more specific indications on how to design the training concept and support options or instructions for patients and family members working with the PEPA (eg, email support, hotline, manuals). With the analysis of the professional perspective, relevant organizational aspect and processes will be taken into account. The research question for this analysis is as follows: What are the potential barriers and necessary requirements for implementation of the PEPA approach in a real-world health care setting? Answering this question includes the following objectives: to (1) point out relevant organizational preconditions for the PEPA implementation and (2) to obtain further results about the necessities for basic support and training courses.

#### Study Design and Methods

The pre-implementation study is based on a mixed methods approach and comprises qualitative and quantitative elements – according to our research aim. We will use a think-aloud method (asking participants to verbalize their thoughts while completing the tasks) for the usability analysis [14]. Additionally, participants will be asked to evaluate their overall satisfaction based on a standardized questionnaire, the System Usability Scale (SUS) [15,16]. For the analysis of preconditions, we will conduct semistructured personal interviews with patients, relatives, HCPs, physicians, and stakeholders.

#### Sample Size

The usability evaluation and the analysis of preconditions are based on 10-15 patients, 10-15 relatives, and 10-15 HCPs as well as physicians. Additionally, we will conduct interviews with up to 20 stakeholders (preconditions of implementation).

#### Recruitment Strategy

Eligible patients will be asked by their responsible physicians in the outpatient clinic of the National Center for Tumor Diseases (NCT) Heidelberg to participate in the study. For recruiting patients’ relatives, all patients participating in the pre-implementation study will also be asked about family members or friends who are supporting them in dealing with the disease. If patients name a certain person, they will be asked to deliver to this person background information about the study.

For recruiting physicians and HCPs at the NCT, we will contact the management of the different professional groups (eg, physicians, nursing staff, social worker, stoma-therapists, nutritionists) and ask for assistance in the recruitment of the participating staff. For recruiting general practitioners and their medical assistants, the Department of General Practice and Health Services Research (GP-HSR; University Hospital Heidelberg) will contact cooperating primary care practices. In addition, representatives of relevant organizations (stakeholder) will be contacted by GP-HSR and asked to participate.

All potential participants will get a written invitation to participate in the study, including background information as well as a declaration of participation and agreement. The written approval for study participation is included in the informed consent document. The signed declaration of participation and agreement must be sent by mail to GP-HSR. There, researchers will contact the participants to arrange an appointment for the usability test and the interview.

#### Inclusion and Exclusion Criteria

To be eligible for participation in the pre-implementation study, patients must have a diagnosis of colorectal cancer (*International Classification of Diseases, Tenth Revision*, ICD-10: C18, C19, C20). The participants must be 18 years of age or older and their disease status has to be classified as Union for International Cancer Control (UICC) stage III-IV. Patients’ relatives do not have to be “related by blood” and could, for example, also be close friends of patients. To be eligible for participation, HCPs have to belong to one of the following groups: clinicians at the NCT, other HCPs such as nursing staff, social worker, stoma-therapists, and nutritionists who are connected to the NCT, as well as general practitioners, according to German regulations, and their medical assistants.

Participating stakeholders should be from organizations such as health insurance funds, large medical centers, medical associations, or political institutions (eg, German Federal Ministry of Health). The sampling of participating stakeholders is based on (1) their thematic interest (2) the position or reputation of the specific stakeholder and (3) the potential impact to foster political decisions for a broader PEPA implementation.

All participants who do not meet the inclusion criteria will be excluded. Additional exclusion criteria for patients are severe acute psychiatric disorders (eg, schizophrenia, schizotypal and delusional disorders); dementia; mental and behavioral disorders due to psychoactive substance use; insurmountable language and communication problems; and emergent cases.

#### Data Collection

The usability evaluation of both portals—patient and professional—aims to simulate activities that should be covered if the PEPA approach is used in real-world health care contexts. Therefore, a test scenario that consists of realistic activities will be developed for the evaluation of the usability. In this test scenario, users will process a multi-item task concerning the functionalities of the patient portal or the professional portal. A think-aloud protocol will be incorporated into the usability test by asking participants to verbalize their thoughts while completing the tasks. After each task, participants will be asked questions about performance and suggestions for improving the system. At the end of the test, participants will be asked to evaluate their overall satisfaction based on the SUS. The SUS allows for calculating a single number representing a composite measure of the overall usability of the PEPA prototype [15]. A German version of the SUS will be used [17]. The whole usability evaluation will be recorded on videotape and should not exceed 60 minutes.

The second part of the pre-implementation study aims to analyze the preconditions of implementation. Therefore, all participants of the usability test are also invited to join a personal interview about potential barriers, ideas for further development, and requirements for transferring the PEPA approach to the care setting. Because external stakeholders will not be participating in the usability test, they will be contacted for these interviews separately. The basis for all interviews will be a semistructured and pilot-tested interview guide. Themes and questions of this interview guide are based on theoretical considerations and findings from a literature review. The interviews will be audiotaped and transcribed verbatim.

#### Data Analysis

Researchers will review and organize qualitative data of the usability test (think-aloud protocols, notes) and the analysis of preconditions (interview transcripts). The qualitative content analysis will include the inductive development of categories and a deductive application of categories. In a first step, transcripts will be reviewed independently by the researchers and key issues will be identified. After summarizing and labeling key issues as codes, these codes will be sorted into main categories and subcategories. The codes will be clearly defined and linked with representative examples from the original texts. After discussing and further modifying all categories within the research team, a consensus on the final category system should be achieved.

To calculate the SUS score, we will first score contributions from each item. In a last step, we will multiply the sum of the scores by 2.5 to obtain the overall value of the SUS [15]. All of these steps (qualitative and quantitative) will be applied in accordance with the particular part of the pre-implementation study and its specific objectives (usability, preconditions).

### Phase 2: Implementation Study

**Figure 3:**
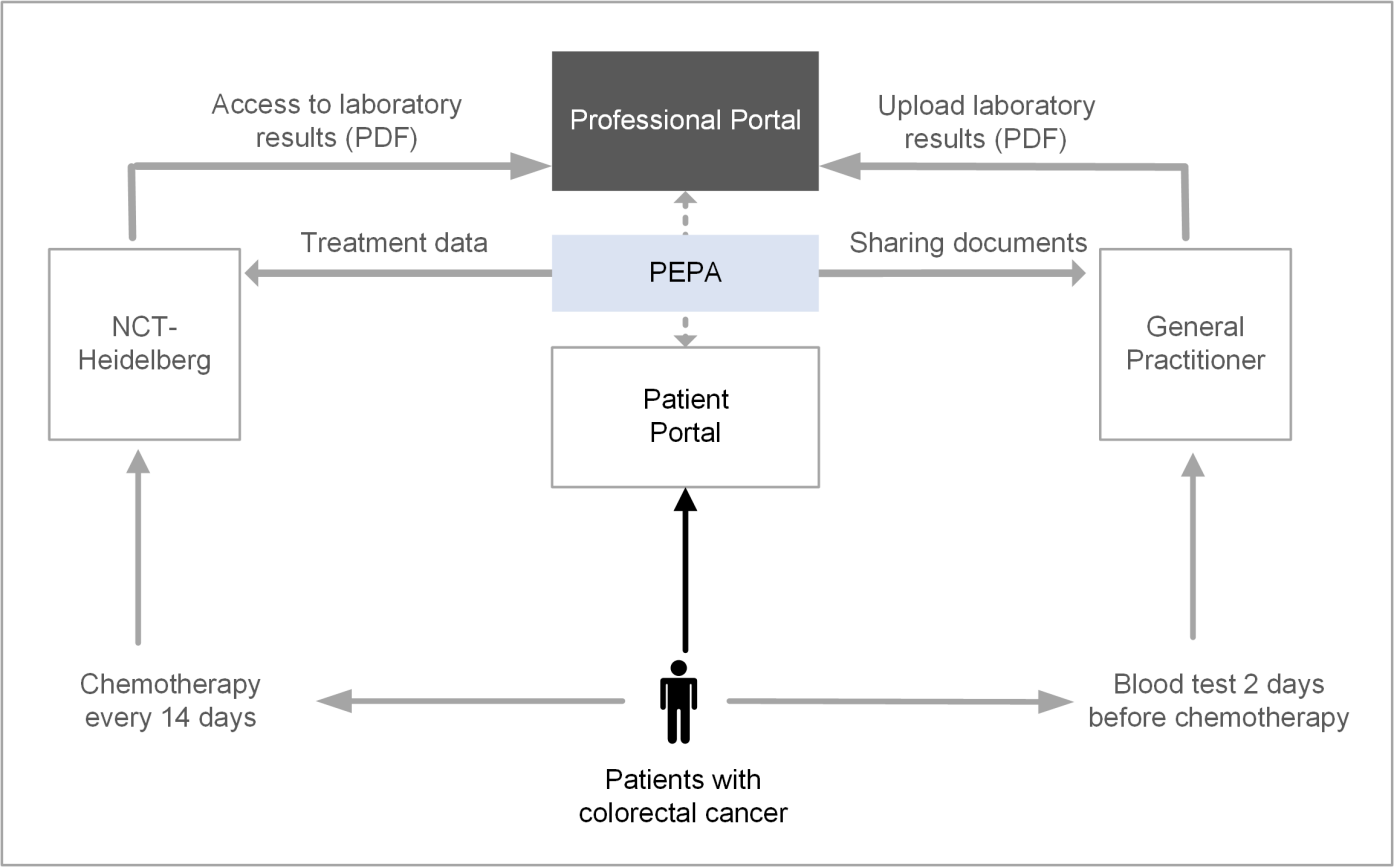
Implementation in a regional care setting. PEPA: personal electronic health record; NCT: National Center for Tumor Diseases.

On the basis of the results of the pre-implementation study, the PEPA prototype will be implemented (only for this study) in a regional care setting. The PEPA implementation aims to give patients access to their treatment documents and to improve processes of care (Figure 3). During the whole study, patients will receive technical and social support.

Within this study, patients have access to their PHI and can also give others (eg, their general practitioner) access to selected or all treatment-related data. In this project, we will use these functionalities to change the process of preparation for chemotherapy. Within the current process, the general practitioner informs the NCT about the latest blood test results via fax 1 day before the patient’s appointment for chemotherapy.

The new PEPA-based process targets the electronic sharing of those blood test results. General practitioners taking part in the study will be able to upload the test results to the PEPA so that the clinicians at the NCT can access the information before the patient arrives for chemotherapy. In addition, the general practitioner will be able to access diagnostic findings and documentation uploaded to the PEPA by the patient or the clinicians at the NCT (Figure 3).

**Figure 4:**
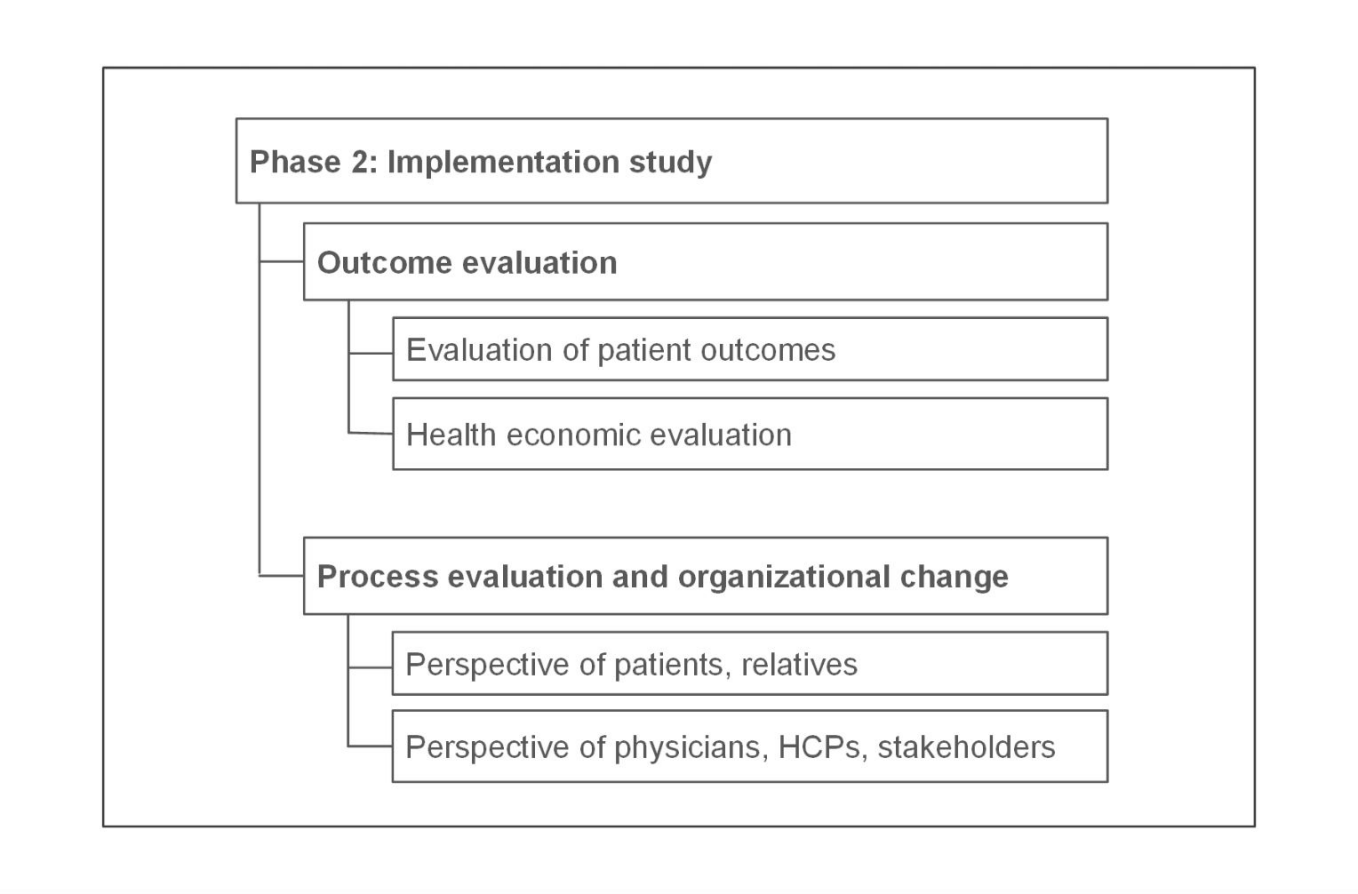
Parts of the implementation study. HCP: health care professional.

All functionalities for general practitioners or clinicians are only possible with permission of the patient. Overall, this feasibility study will consider how patient outcomes can be improved and processes can be changed through the implementation of the PEPA approach. This study comprises 2 parts: outcome evaluation as well as process evaluation and organizational change (Figure 4).

#### Objectives

##### Outcome Evaluation

The primary objective of this study is to assess patients’ self-efficacy, among all participants, using the German version of the Cancer Behavior Inventory–Brief Version (CBI-B-G). CBI-B-G has high internal consistency (Cronbach alpha=.85) and correlates substantially with generalized self-efficacy (*r*=.57, *P*<.001) and emotional functioning (*r*=.53, *P*<.001) [18].

CBI-B-G scores are assessed at baseline (T0) and after 12 weeks of PEPA usage (T1). We will determine whether there is a difference in CBI-B-G scores after using the PEPA prototype for 3 months (T1) compared with baseline (T0). Furthermore, a number of secondary outcomes will be measured (see Table 1).

**Table 1:** Overview of outcome parameters and measurement instruments in this study.

Outcome parameters	Measurement instruments	Items
Patient self-efficacy (primary outcome)	German version of the Cancer Behavior Inventory–Brief Version (CBI-B-G) [18]	14
Control preferences	Control Preferences Scale [19]	5
Psychosocial distress	Distress Thermometer [20]	1
Utilization of medical services	[Mannheimer Module Resource Consumption]^a^	30
Involvement in care	Perceived Involvement in Care Scale [21]	13
Usability of PEPA^b^ prototype	System Usability Scale [15]	10

^a^Not published.

^b^PEPA: personal electronic health record.

In terms of health economic evaluation, it has been hypothesized that PHRs increase the virtual access to care and reduce health care costs [22,23]. The objective of this health economic work package is to collect data likely to support or reject these assumptions. This includes the following:

- to collect data on service utilization and unit cost of treatments or service contacts in order to assess direct medical costs of study subjects 3 months before the intervention and during the PEPA-intervention phase
- to collect data on work absentees and productivity loss 3 months before the intervention and during the PEPA-intervention phase to estimate indirect costs of study subjects
- to identify potential factors and PEPA-related effects likely to influence health care costs from the health system and societal perspective

##### Process Evaluation and Organizational Change

The aim for this analysis is to understand which factors promote or hinder the implementation of the PEPA approach in the defined care setting (see Figure 3). With the process evaluation, we will focus on the identification of facilitators and barriers within the implementation process. Relevant in this context is how the PEPA implementation may affect health care organizations and lead to the need for organizational change. Additionally, we will specify relevant change management standards for a successful implementation of the PEPA approach in health care organizations.

Research questions for this analysis are as follows: Is it feasible to implement and use the PEPA approach under real-world health care conditions? What are the relevant change management strategies that are able to support successful implementation process in health care organizations? Answering these questions includes the following objectives: to (1) track relevant barriers and facilitators of the implementation and (2) create relevant change management standards for health care organizations.

#### Study Design and Methods

The outcome evaluation is planned as a prospective, 3-month, open-label “before and after” trial. Additionally, for the analysis of processes and organizational change, we will conduct interviews with general practitioners, HCPs, physicians, and patients who are participating in the before and after trial.

#### Sample Size

For the planned before and after trial, the sample size of up to 30 patients is solely based on matters of feasibility (due to the exploratory nature of the trial). With this number of patients, a standardized treatment effect of 0.53 can be demonstrated with a power of 1−β=0.8 at a (descriptive) two-sided significance level of alpha=.05 by applying a paired-sample *t* test. Additionally, we will conduct interviews with all participating patients and professionals.

#### Recruitment Strategy

Physicians and HCPs will be contacted directly. Potential participants will get a written invitation to participate in the study, including background information for physicians and other HCPs as well as a declaration of participation and agreement. The written approval for participation in the study is included in the informed consent document. The potential participants will send their declaration and participation agreement by mail to GP-HSR. All clinicians at the NCT using the PEPA will receive training demonstrating how to deal with this new instrument.

All patients matching the inclusion criteria will be contacted by their responsible physician at the NCT and asked to participate in the study. The responsible physician will inform them about aims, content, privacy issues, and risks related to the study. After patients give their consent to participate in the study, they will receive a pseudonym. Subsequently, patients will be invited to come to the GP-HSR and receive comprehensive training for handling the PEPA. This training includes a complete introduction to the functionalities of and consequences of working with the PEPA (eg, data security issues, allocating access authorizations). Then the PEPA will be set up individually for every patient, including the transfer of existing PHI (eg, former findings) to the PEPA (via PDF upload).

On the basis of the care setting of the intervention, the general practitioners (and their medical assistants) will be recruited depending on the selected patients. The corresponding general practitioners of the selected patients will be contacted and asked to participate in the study by GP-HSR. With an existing interest, the potential participants will get a written invitation to participate in the study, including background information for physicians and other HCPs as well as a declaration of participation and agreement. Causes for nonparticipation will be documented by GP-HSR. The informed consent document contains the request to give their written approval for participation in the study. The potential participants will send their declaration and participation agreement by mail back to GP-HSR. All participating general practitioners and their medical assistants will receive PEPA training.

#### Inclusion and Exclusion Criteria

Patients must have a diagnosis of colorectal cancer to be eligible for participation (ICD-10: C18, C19, C20). Furthermore, they should be receiving either chemotherapy with curative approach at the NCT after their primary surgery (at least for the next 2 months) or chemotherapy after relapse with symptom-relieving approach at the NCT. The participants must be 18 years of age or older and their disease status has to be classified as UICC stage III-IV.

To be eligible for participation in the study, the clinicians and other HCPs of the NCT as well as general practitioners and their medical assistants have to cooperate closely with the included patients during their treatment at the NCT. Prerequisite for inclusion of patients and professionals in analysis of processes and organizational change is participation within the before and after trial.

All participants who do not meet the inclusion criteria will be excluded. Additional exclusion criteria for patients are severe acute psychiatric disorders (eg, schizophrenia, schizotypal and delusional disorders); dementia; mental and behavioral disorders due to psychoactive substance use; insurmountable language and communication problems; and emergent cases.

#### Data Collection

Data will be collected from patient survey responses and shall be obtained for all patients. Additionally, a sociodemographic questionnaire will help to gain information on age, sex, diagnoses, and educational level. Patients will receive a paper-based outcomes survey questionnaire right before the intervention starts (baseline) and at the end of the 3-month test period. The completion of each questionnaire will take about 45 minutes. All data will be pseudonymized. The collected data will be entered into a database and stored on a secured server.

The basis for conducting interviews will be a semistructured and pilot-tested interview guide. Each interview will be conducted until no newer aspects will be addressed. The interviews will be performed by a trained researcher (the moderator). All interviews will be audio- and videotaped and transcribed verbatim. Videotapes will be used to assist with the transcription of group data. Additionally, sociodemographic data will be collected anonymously using a study-specific questionnaire.

The health economic evaluation focuses on patients and their health services utilization. All consumed goods and services will be assessed from the societal perspective. This perspective assures that all relevant cost categories are included. Service utilization and intervention-related costs are measured by an adopted version of the “Mannheimer Module Resource Consumption” questionnaire. The consumed resources are weighted by standardized unit costs to derive direct and indirect costs.

#### Data Analysis

Because of the exploratory nature of the trial, the primary outcome “self-efficacy”—captured by the CBI-B-G [18]—will be evaluated descriptively at time points T0 and T1, by tabulating the respective means, SDs, medians, first and third quartiles, and minimum and maximum. Furthermore, descriptive *t* tests for paired samples will be applied to investigate potential differences between time points T0 and T1 and descriptive *P* values and 95% confidence intervals will be given. Missing values for the primary outcome at T1 will be replaced by multiple imputation [24] taking the baseline value (T0) into account. Best- and worst-case imputation will be conducted as sensitivity analyses. As in the case of the primary outcome, all secondary outcomes (see Table 1) will be analyzed descriptively by tabulating the measures of the empirical distributions. For continuous outcomes, means, SDs, medians, first and third quartiles, and minimum and maximum will be provided. For categorical outcomes, absolute and relative frequencies will be reported.

For health economic evaluation, descriptive analysis of the excess costs related to the participating patients will be scrutinized. Standard measures of central tendencies and dispersions are selected. This type of cost-of-illness study yields empirical insights into costs and cost components of the PEPA under real-world conditions.

In terms of process evaluation and organizational change, the transcribed texts of all interviews will be the basis for performing the qualitative content analysis. Data will be taken from the transcribed texts, edited, and analyzed [25]. This will be done by using a preliminary category system (search grid), which is based on themes and questions of the interview guide. In addition, the category system will be continuously adapted during the analysis process. For data analysis, in a first step, 2 out of all transcriptions will be analyzed independently by 3 members of the research team to identify relevant key issues. Following that, the key findings will be discussed within the research team and the preliminary category system will be adapted. Afterward, all key issues will be labeled as codes and these codes will be organized into main categories and subcategories. Each code will be clearly defined and linked with samples from the transcriptions. Labeling categories will be performed by using ATLAS.ti version 7.0.80 (Scientific Software Development GmbH).

#### Development of a Training Program

On the basis of the investigations that will be made in the other study parts, a training program will be developed. It is a planned by-product that characterizes one essential element for a successful future implementation of the PEPA in a real-world health care setting. The aspired function of the training concept is to cover the demands of the patients, family members, and HCPs for support required for using the PEPA in their daily routine. All patients and staff members using the PEPA prototype will receive training demonstrating how to deal with this new instrument.

#### Ethical Consideration

The pre-implementation study as well as the implementation study will be conducted in accordance with medical professional codex and the Declaration of Helsinki (2013). The study is also in accordance with German Federal Data Protection Act (BDSG). All professionals participating in the study are obliged to adhere to the abovementioned declarations and laws. Participation for patients and HCPs is voluntary. Consent can be withdrawn at any time without any consequences for patients’ (usual) care. If a patient withdraws his or her consent, data that have already been collected can either be destroyed upon request of the respective patient (if the data have not been included in an already published work) or will be analyzed if he or she agrees. All patients will be informed about aims, content, duration, and process of the trial, particularly as far as risks and unintended consequences are concerned, through written information brochures and through face-to-face communication with staff of the study central office and with the responsible physician at the NCT. All collected data (eg, questionnaires, audio- and videotapes) will be saved according to applicable laws and regulations and afterward irretrievably deleted.

This study protocol was approved by the Ethics Committee of the Medical Faculty of the University of Heidelberg (S-462/2015).

## Results

This project is part of the INFOPAT project, which is funded (2012-2016) by the Federal Ministry of Education and Research (BMBF). The enrolment was completed in July 2016. Data analysis is currently under way and the first results are expected to be submitted for publication at end of 2017.

## Discussion

### Summary

If health care is provided in more than one care setting or health system, the availability of treatment-related data within the process of care and the option for patients to manage their own PHI are limited. This may have consequences for the efficacy of treatment and options for the engagement of patients within their treatment (self-management). The development of the PEPA approach aims to address these problems. However, with this study and for the first time we will implement the PEPA approach in a real-world health care setting. Existing approaches of PHRs aim to give patients access to their treatment data. With this study, we go a step further: patients have access and they can also give other persons (eg, their general practitioner) access to their treatment data. With this approach, new possibilities of collaboration between different providers and for the engagement of patients may arise. However, study design and sample size are based on pragmatic considerations and closely related to challenges of the PEPA implementation in a real-world health care setting. In this way, the transferability of our study results may be limited.

### Strength and Limitations

One major strength of this feasibility study is that we do not focus only on outcomes. Instead, we are taking the whole process of implementation into account. This means that we will start with a usability evaluation and the consideration of organizational preconditions for the implementation of the PEPA approach (pre-implementation study). Additionally, we will evaluate the process of implementation (eg, barriers or facilitators), the need for organizational change (eg, processes of communication), and the impact on outcomes (eg, self-efficacy) within the implementation study.

However, this study has a number of limitations. The implementation of the PEPA approach in this study is focused on patients with colorectal cancer. Conclusions for other chronic diseases may not be conceivable. Additionally, the evaluation of outcomes is based on a before and after trial, with a small number of participants and only 3 months of exploration. Causal correlations cannot be explained with this approach as the evaluation of outcomes is only explorative.

In terms of risks and (unintended) effects, for the participation in focus group discussions or guided interviews no severe or unexpected adverse events are mentioned within the literature. Nevertheless, it has to be kept in mind that the participants could feel uncomfortable within the discussion or interview setting. Furthermore, the participation could strengthen already established misgivings concerning the use of innovative health information technology. Additionally, it is possible that patients using the PEPA and the included complex information get a much deeper look into their PHI, which could lead to arising uncertainty or feelings of being overloaded.

## Abbreviations

BMBF: Federal Ministry of Education and Research
CBI-B-G: Cancer Behavior Inventory–Brief Version (German version)
GP-HSR: Department of General Practice and Health Services Research
HCP: health care professional
ICD-10: International Classification of Diseases, Tenth Revision
INFOPAT: information technology for patient oriented care
NCT: National Center for Tumor Diseases
PEPA: personal electronic health record
PHI: personal health information
PHR: personal health record
SUS: System Usability Scale
UICC: Union for International Cancer Control
